# Investigation of the Deactivation and Reactivation Mechanism of a Heterogeneous Palladium(II) Catalyst in the Cycloisomerization of Acetylenic Acids by *In Situ* XAS 

**DOI:** 10.1021/acscatal.0c04374

**Published:** 2021-02-22

**Authors:** Ning Yuan, Arnar Gudmundsson, Karl P. J. Gustafson, Michael Oschmann, Cheuk-Wai Tai, Ingmar Persson, Xiaodong Zou, Oscar Verho, Éva G. Bajnóczi, Jan-E. Bäckvall

**Affiliations:** † Department of Materials and Environmental Chemistry, Arrhenius Laboratory, Stockholm University, SE-106 91 Stockholm, Sweden; ‡ Department of Organic Chemistry, Arrhenius Laboratory, Stockholm University, SE-106 91 Stockholm, Sweden; § Department of Molecular Sciences, Swedish University of Agricultural Sciences, P.O. Box 7015, SE-750 07 Uppsala, Sweden; ∥ Department of Medicinal Chemistry, Uppsala Biomedical Centre, Uppsala University, SE-751 23 Uppsala, Sweden; ⊥ Wigner Research Centre for Physics, H-1121 Budapest, Hungary; # Department of Natural Sciences, Mid Sweden University, Holmgatan 10, SE-851 70 Sundsvall, Sweden

**Keywords:** X-ray absorption spectroscopy, cycloisomerization, deactivation/reactivation, heterogeneous, palladium catalysis

## Abstract

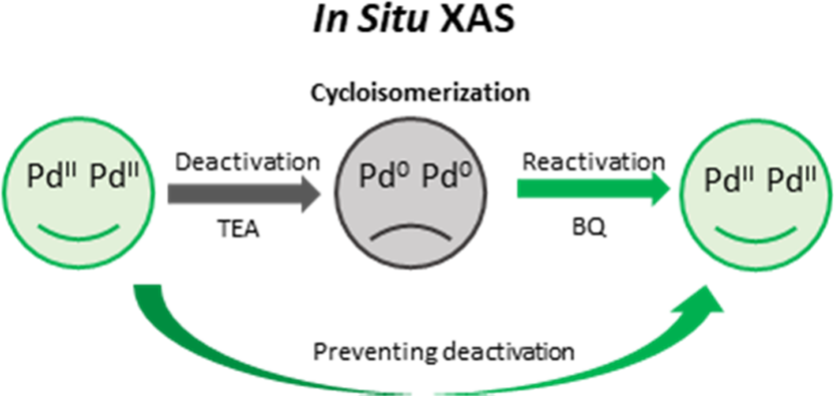

A well-studied heterogeneous palladium(II) catalyst used for the cycloisomerization of acetylenic acids is known to be susceptible to deactivation through reduction. To gain a deeper understanding of this deactivation process and to enable the design of a reactivation strategy, *in situ* X-ray absorption spectroscopy (XAS) was used. With this technique, changes in the palladium oxidation state and coordination environment could be studied in close detail, which provided experimental evidence that the deactivation was primarily caused by triethylamine-promoted reduction of palladium(II) to metallic palladium nanoparticles. Furthermore, it was observed that the choice of the acetylenic acid substrate influenced the distribution between palladium(II) and palladium(0) species in the heterogeneous catalyst after the reaction. From the mechanistic insight gained through XAS, an improved catalytic protocol was developed that did not suffer from deactivation and allowed for more efficient recycling of the catalyst.

## Introduction

Over the past few decades, transition metal catalysis has played a key role in advancing the field of organic chemistry by enabling a wide range of transformations that would not have been possible to achieve through classical chemical means.^1,2^ Here, transition metal-based catalytic protocols have been absolutely instrumental in introducing fundamentally new ways of forming bonds and assembling molecules. When designing a new catalytic protocol or improving an existing one, it is important to have a clear understanding of the underlying reaction mechanism as this knowledge is essential for an efficient catalyst design. As a result, mechanistic studies have become an increasingly more common element in methodology development over the years. Traditionally, it has been proved to be significantly more challenging to study the mechanism of catalytic species on a surface compared to those present in solution, mostly because the analytical techniques available for heterogeneous reactions are fewer and less accessible.

X-ray absorption spectroscopy (XAS) is an analytical technique that has gained particular popularity in recent years. It is an element-specific technique that can be used *in situ* to directly probe the nature of catalytic species in real time and to monitor how they change over the course of a reaction. Many gas–solid heterogeneous catalytic systems have been studied by *in situ* XAS, providing valuable insights into their reaction mechanisms.^3−10^ However, traditionally, it has been difficult to obtain *in situ* XAS data for catalytic systems in suspension or solution due to the challenges associated with the reactor design and the relatively strong interferences of the reaction mixture on X-rays. Fortunately, thanks to recent progress in the field, several successful studies have been reported where the nature of the catalytic species has been elucidated through *in situ* XAS.^11−25^

In our laboratory, we have been interested in the transition-metal-catalyzed cycloisomerization of acetylenic acids,^26−28^ since this reaction provides easy access to the γ-alkylidene lactone motif, which is a structural element present in many biologically active natural products. Various protocols based on different transition metals such as Ru,^29^ Rh,^30,31^ Au,^26,32^ and Pd^27,33−36^ have been developed for this transformation, but many of them require harsh reaction conditions, long reaction times, and/or high catalyst loading. In addition, most of these reports involve homogeneous catalysts, which can be difficult to separate and recycle, although a few examples of heterogeneous catalysts do exist.^26,27,35,37^ Recently, we developed heterogeneous catalysts where palladium is immobilized on aminopropyl(AmP)-functionalized siliceous mesocellular foam (MCF), which have been applied in various transformations.^27,38−41^ In some of these catalysts, Pd(II) was immobilized on this carrier leading to Pd(II)-AmP-MCF^27,40,41^ and we reported its use in the cycloisomerization of acetylenic acids.^27^ Although Pd(II)-AmP-MCF proved to be a highly efficient catalyst for this transformation, it was discovered to suffer from considerable deactivation over repeated cycles. In the recycling study using pent-4-ynoic acid as the substrate, catalyst deactivation was already observed in the first cycle despite leaching being negligible. We proposed that the cause of this deactivation was the formation of catalytically inactive Pd(0) species. Interestingly, we found that the activity of the catalyst could be restored upon treatment with benzoquinone (BQ),^27^ which presumably coordinates to palladium in the heterogeneous catalyst and triggers a reoxidation of the inactive Pd(0) to active Pd(II). However, so far this mechanism has not yet been experimentally confirmed. In the present study, we provide the first evidence for the previously proposed mechanism by the use of XAS. Furthermore, the broader mechanistic understanding that we gained through the use of XAS allowed us to develop an improved reactivation strategy, which enabled for highly efficient recycling of the catalyst.

Herein, we present a convincing example of how XAS can be utilized to support a mechanistic investigation and to enable the optimization of a catalytic system. The results from our mechanistic studies of two model cycloisomerization reactions using *in situ* XAS give a clear picture of the deactivation process. These results are presented alongside the newly devised reactivation strategy and recycling studies. A customized *in situ* reactor (Figure S1, Supporting Information) was used to study the catalytic reactions, which allowed for the XAS data to be collected continuously.

## Results and Discussion

**Scheme 1:**
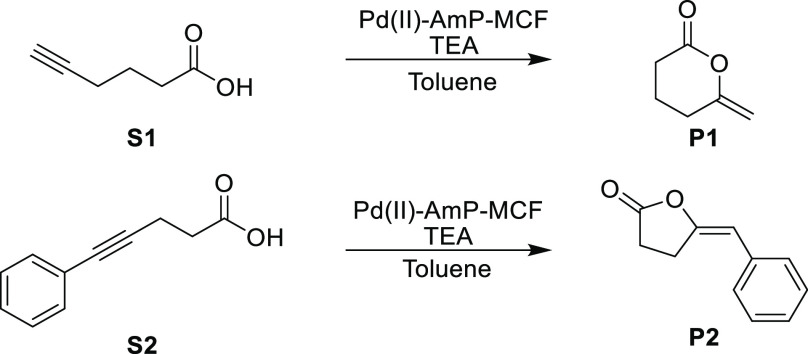
Cycloisomerization of Acetylenic Acids to Lactones Catalyzed by Pd(II)-AmP-MCF

The local structure of the Pd centers in Pd(II)-AmP-MCF was established in a previous study using the extended X-ray absorption fine structure (EXAFS) region of the XAS spectrum.^42^ In this previous study, each Pd center was found to bind two aminopropyl groups of the catalyst support and two chloride ligands. The presence of these chloride ligands can be explained as originating from the Pd precursor (Li_2_PdCl_4_), which is used in the synthesis of Pd(II)-AmP-MCF to introduce Pd into the nanoparticle scaffold. In the present study, Pd(II)-AmP-MCF was used in model reactions to convert hex-5-ynoic acid (**S1**) and 5-phenylpent-4-ynoic acid (**S2**) to their corresponding lactones, in the presence of triethylamine (TEA) (Scheme 1).

**Figure 1:**
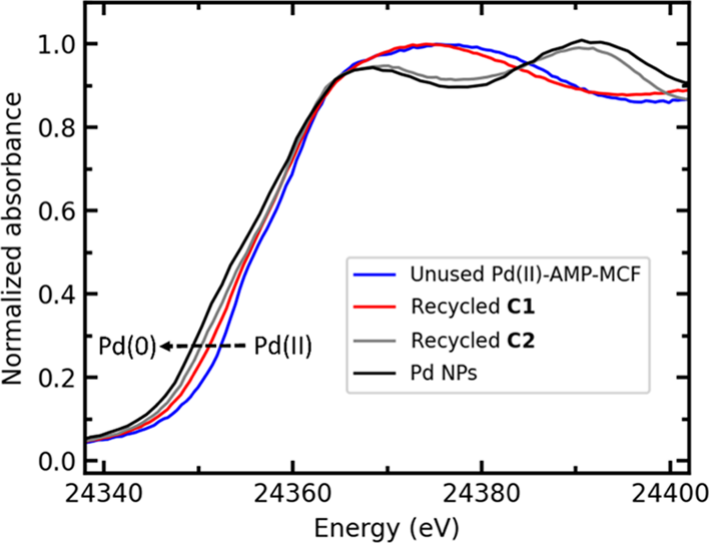
*Ex situ* Pd K-edge XANES spectra of unused Pd(II)-AmP-MCF, recycled **C1** and **C2**, and the Pd NP reference. The Pd NP reference data is taken from ref (43).

The catalysts recycled after one reaction cycle with **S1** (hereafter labeled recycled **C1**) and **S2** (hereafter labeled recycled **C2**) were first measured by XAS in an *ex situ* manner to determine the nature of the Pd species. Figure 1 compares the X-ray absorption near edge structure (XANES) region of the XAS spectra of unused Pd(II)-AmP-MCF and the recycled **C1** and **C2**, along with a sample of Pd nanoparticles (Pd NPs) as a Pd(0) reference. It is worth mentioning that the XAS data of the Pd NPs with particle sizes of around 2 nm was collected under a helium flow to prevent potential oxidations.^43^ Interestingly, Figure 1 shows only a minor change of the recycled catalyst **C1** compared to the unused catalyst. On the other hand, recycled **C2** displays XANES features that resembled those of the Pd NPs. The positions of the absorption edges of the recycled catalysts are located between the unused catalyst and the Pd NPs. These observations together suggest that at least a partial reduction of the recycled catalysts had occurred, which was more extensive in the catalytic reaction with **S2** than that with **S1**, indicating that the choice of the substrate has a substantial effect on the reduction of Pd.

**Figure 2:**
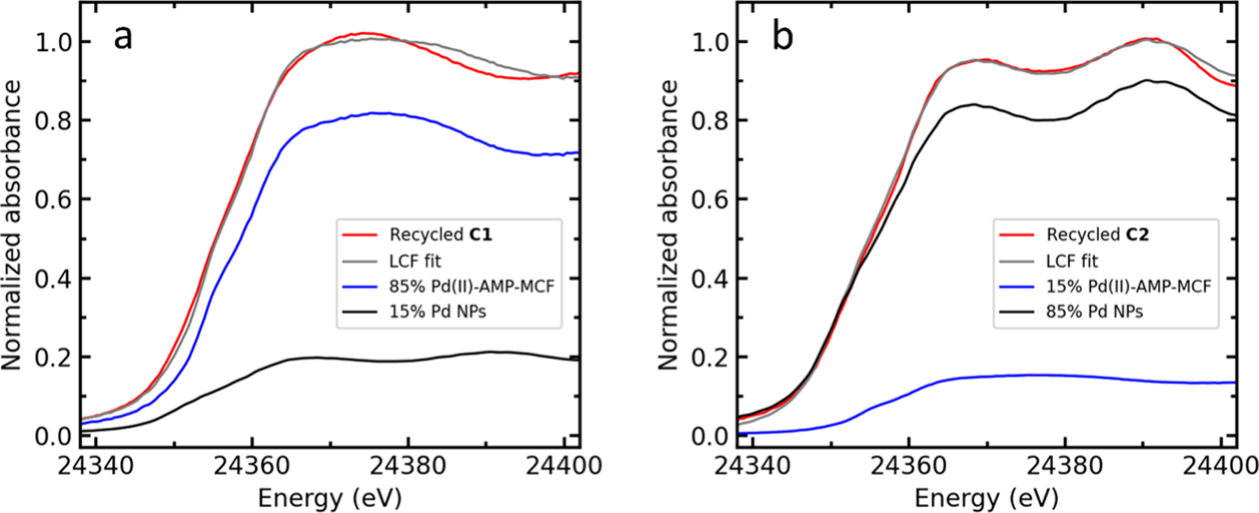
Linear combination fit of Pd K-edge XANES spectra of recycled (a) **C1** and (b) **C2**. The XANES spectra of the Pd NPs and unused Pd(II)-AMP-MCF were used as the references for the metallic Pd component and the Pd(II) complex, respectively. All of the spectra used for the LCF are normalized and calibrated. Different weights of the references are iterated to find the best fit, while the sum of weights is always fixed at 1 and the *E*_0_ is not iterated in the fitting.^12,44^

A linear combination fit (LCF) of the XANES spectra was then applied to estimate the fractions of metallic and unreduced species, as shown in Figure 2. The XANES spectra of the Pd NPs and unused Pd(II)-AMP-MCF were used as the references for metallic Pd(0) species and Pd(II) species, respectively. The reason Pd NPs are preferred over Pd foil to perform a LCF is that the XANES spectrum of bulk Pd has a larger amplitude than that of Pd NPs^12,44^ as the number of Pd–Pd distances per palladium decreases, as well as the mean Pd–Pd bond distances, with decreasing particle size. A comparison of their XANES spectra can be seen in Figure S2, which clearly shows the amplitude differences. Meanwhile, the edge positions of Pd foil and Pd NPs overlap well, which confirms that the oxidation state of the Pd NPs is 0.

The XANES spectrum of recycled **C1** could not be fitted very well using the current references, which indicates the existence of other Pd species, likely in the form of Pd(N/O)_4_-type coordination. This hypothesis was supported by principal component analysis (PCA), which suggests that likely at least three different components are necessary to describe the speciation. The screen-plot of the PCA analysis is shown in Figure S3 (Supporting Information). However, the LCF results still revealed that only *ca*. 15% of Pd atoms are in the form of nanoparticles and that the majority of the Pd is in the form of the Pd(II) complex. Interestingly, the XANES spectrum of recycled **C2** could be fairly well fitted by 85% Pd NPs and 15% unused catalyst, although a negligible amount of a third Pd species with a similar coordination environment to metallic Pd or Pd(II)-AmP-MCF might be present as well according to its PCA analysis (Figure S3). These results indicate that Pd(II) has to a large degree been reduced into Pd(0) nanoparticles during the reaction.

**Figure 3:**
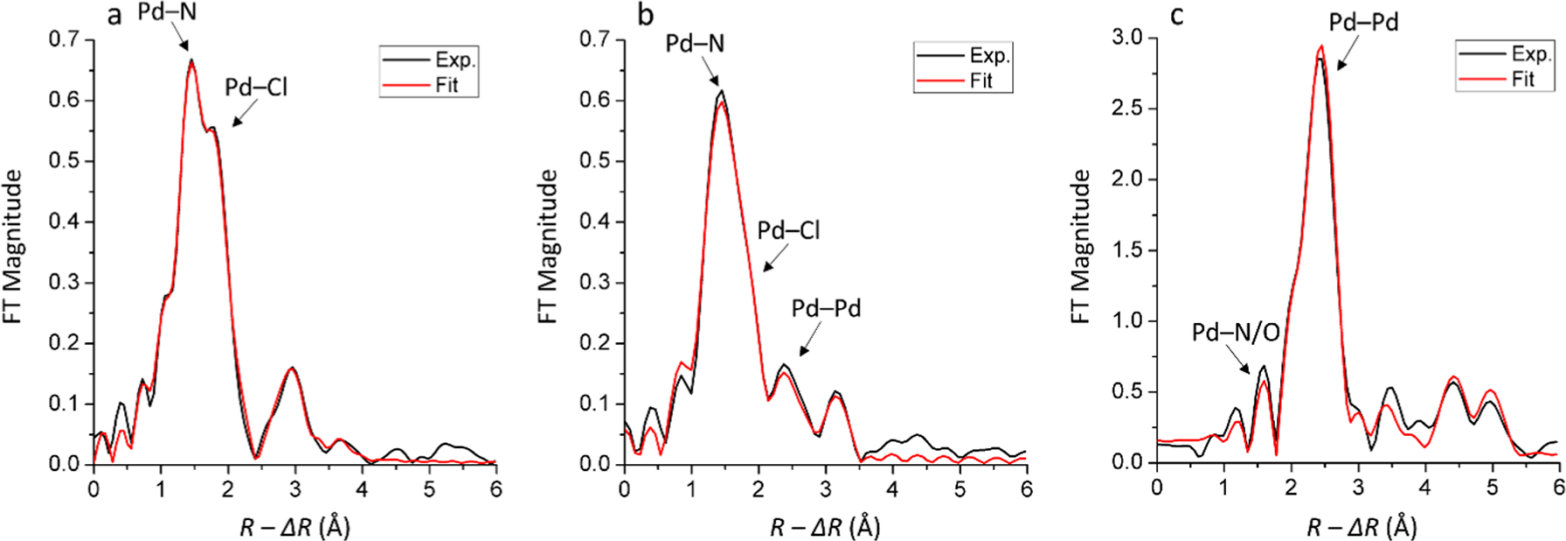
Fourier transformed *k*^3^-weighted EXAFS spectra of (a) unused Pd(II)-AmP-MCF and the recycled catalysts (b) **C1** and (c) **C2**. The spectra are not phase-corrected. The *k* ranges used to perform Fourier transform are 2–13, 2–10, and 2–12 Å^–1^, respectively.

To further elucidate the nature of the recycled Pd species **C1** and **C2**, their EXAFS spectra were analyzed. Figure 3 shows the Fourier transformed EXAFS data, and the refinement parameters of the first coordination shell are summarized in Table 1. In the unused catalyst, two peaks corresponding to Pd–N and Pd–Cl are the dominating contributions. Single scatterings of Pd···Pd at distances longer than the Pd–Pd bond are also observed, and they explain the peaks at around 3 Å in Figure 3a (Table S2). For the recycled catalyst **C1**, only one dominant peak at the position matching Pd–N/O is present (Figure 3b). About two N/O–ligands on average were found to bind to each Pd atom with a mean distance of 2.03 Å. The bond distance, *d*, and Debye–Waller coefficients, σ^2^, are found to be similar to those of the unused catalyst, indicating that the aminopropyl ligand bound to Pd in the unused catalyst is most likely retained in recycled **C1**. Meanwhile, the average coordination number (**CN**) of Pd–Cl decreases to 0.8 in recycled **C1** (Table 1), and its signal appears as a subtle shoulder on the main peak at *ca*. 1.8 Å (without phase correction) in Figure 3b. The peak at *ca*. 2.4 Å (without phase correction) in Figure 3b is fitted by Pd–Pd single scattering in metallic Pd and the average coordination number (**CN**) of such distances is 0.5 (Table 1). Pd···Pd single scatterings corresponding to the second and third shells of the crystalline structure of metallic Pd are observed in recycled **C1** and **C2**. The refined structure parameters are summarized in Table S2. Overall, the Pd centers in recycled **C1** appear to be primarily coordinated to amino ligands, with a small fraction being involved in metallic Pd aggregates. However, a significant amount of Cl^–^ ligands was detached from the Pd centers compared to the unused catalyst. Due to the formation of Pd aggregates and the potential surface oxidation, Pd–O bonds are expected to be present as well. Species containing Pd–O bonds cannot be distinguished from those with Pd–N bonds by the current EXAFS data because of the very similar bond distances and the back-scattering ability of such species. However, the presence of Pd–O bonds is supported by the observations from the LCF and PCA analyses of its XANES spectrum, as discussed above. On the other hand, in recycled **C2**, only one main peak is observed and it is refined to a distance of 2.74 Å, which can be ascribed to metallic Pd aggregates.^11^ In contrast, the contribution from Pd–N/O was found to be very minor in recycled **C2**. To clarify if the peak at *ca*. 1.6 Å (without phase correction) contains a real signal of Pd–N/O, a comparison of the fitting with and without introducing Pd–N/O single scattering is shown in Figure S6 (Supporting Information). The fit of this peak improves when the Pd–N/O single scattering is included, which suggests a real chemical contribution.^45^ This analysis agrees with the LCF discussion above revealing a small fraction of a Pd complex. Additionally, its edge position is slightly above that of Pd NPs, which also indicates the existence of a small fraction of Pd species with a higher oxidation state. This minor signal could be from a small amount of the remaining Pd(II) complexes or from the oxidized surface of the Pd aggregates. Overall, these observations agree well with the preliminary analysis of the XANES spectra.

**Table 1:** Coordination Number, **CN**, Mean Distances, *d* (Å), Debye–Waller Coefficients, σ^2^ (Å^2^), and Many-Body Amplitude Reduction Factor, *S*_0_^2^ in the EXAFS Studies of the Catalyst at Different Conditions^a^

samples	interaction	CN^b^	*d* (Å)	σ^2^ (Å^2^)	*S*_0_^2^
Pd(II)-AmP-MCF	Pd–N	2.0	2.023(2)	0.0035(3)	0.93(2)
	Pd–Cl	2.0	2.294(2)	0.0058(2)	
recycled C1	Pd–N/O	2.0	2.034(4)	0.0032(7)	0.93(5)
	Pd–Cl	0.8	2.337(5)	0.0035(9)	
	Pd–Pd	0.5	2.72(1)	0.011(2)	
recycled C2	Pd–N/O	0.5	2.07(3)	0.004(3)	0.92(4)
	Pd–Pd	8.0	2.741(2)	0.0062(2)	
catalyst before addition of BQ (S2)	Pd–N/O	1.0	2.05(1)	0.003(2)	0.92(8)
	Pd–Pd	7.0	2.734(3)	0.0042(6)	
catalyst after addition of BQ (reactivation, S2)	Pd–N/O	0.6	2.05(4)	0.002(6)	0.9(1)
	Pd–Cl	0.6	2.35(3)	0.004(4)	
	Pd–Pd	7.0	2.726(5)	0.0064(8)	
catalyst after addition of BQ (prevention of deactivation, S2)	Pd–N/O	1.5	2.011(8)	0.002(2)	0.84(7)
	Pd–Cl	1.5	2.303(6)	0.003(1)	
	Pd–Pd	1.5	2.731(6)	0.0075(7)	

a The standard deviations in parentheses were obtained from *k*^3^-weighted least-squares refinement of the EXAFS function χ(*k*) and do not include systematic errors of the measurement.

b The estimated error of the average coordination number (CN) is *ca*. 25%. The true CN is the average CN divided by its corresponding fraction. Underscored parameters were optimized from several trials and were fixed in the individual refinements. The fitting of the corresponding EXAFS spectra and their Fourier transformations are presented in Figures S4 and S5, respectively. The values of reduced error (χ^2^), Δ*E*_o_, and *k*-ranges of the fittings are presented in Table S1. Parameters of all scattering paths including outer coordination shells and multiple scattering can be found in Table S2 (Supporting Information).

**Figure 4:**
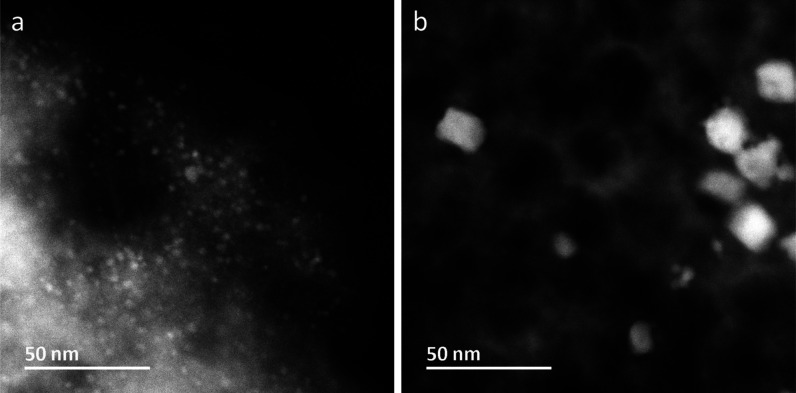
High-angle annular dark-field (HAADF) STEM images of recycled **C1** (a) and **C2** (b) showing the Pd nanoparticles (in white).

Scanning transmission electron microscopy (STEM) was also carried out to investigate the presence of any Pd nanoparticles in the recycled catalysts (Section S4, Supporting Information). Here, Pd nanoparticles were found in both recycled **C1** and **C2**, as shown in Figure 4a,b, respectively. This observation is consistent with the XAS data that showed the formation of metallic Pd species in both cases. STEM images also show a large size variation as well as aggregations of Pd nanoparticles (Figures 4 and S11, Supporting Information). Nevertheless, it is obvious that the overall size of the Pd nanoparticles in recycled **C1** was much smaller than those of recycled **C2**. The difference in Pd particle size can also be correlated to the mean bond length of Pd–Pd as determined by EXAFS (see Table 1), since the mean Pd–Pd bond distance shortens with decreasing particle size as a result of the larger proportion of surface atoms that have lower coordination numbers than the interior ones.^12,46^ In the case of **C2**, the average Pd–Pd coordination number is *ca*. 8.0. Considering that its fraction is 85%, the true coordination number can be deduced to be *ca*. 9.5, which corresponds to a particle size of ≥4 nm.^12^ It is also evident from the EXAFS data in Figure 3b that Pd aggregates (Pd(0)) were the minor Pd species in recycled **C1**, meaning that the majority of the Pd species exist in other forms than the nanoparticles. It should be noted that molecular Pd species are not visible in the STEM images and the distribution of the Pd nanoparticles in the AmP-MCF support is not uniform (Figures 4 and S11, Supporting Information); it is therefore not possible to make an estimation of the fraction of Pd particles in the samples based on the STEM images.

**Figure 5:**
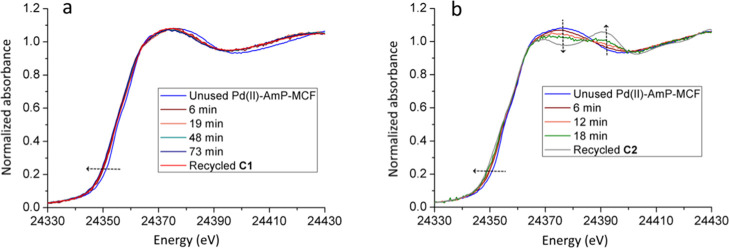
Representative *in situ* Pd K-edge XANES spectra of Pd(II)-AmP-MCF used in catalytic cycloisomerization of **S1** (a) and **S2** (b).

To understand the changes to the Pd centers during the reactions, *in situ* XAS measurements were performed using **S1** and **S2** individually with unused Pd(II)-AmP-MCF. The stability of Pd(II)-AmP-MCF in toluene as solvent was first investigated before the start of the reaction. Figure S7 (Supporting Information) shows that the XANES spectra of unused Pd(II)-AmP-MCF in dry conditions and suspended in toluene are identical, meaning that no changes occur when the catalyst is suspended. After excluding any influence by the solvent, the catalytic reactions were initiated and the XAS spectra were collected every 6 min. Figure 5 shows representative *in situ* XANES spectra for the catalytic reactions with **S1** and **S2**. Immediate changes in the first *in situ* XANES spectra (6 min) occurred for the reactions of both substrates. However, in the case of the reaction with **S1**, the *in situ* XANES spectra remained unchanged after this initial change and matched the *ex situ* spectrum of recycled **C1** very well (Figure 5a). This means that the Pd speciation during the measurement of the reaction **S1** → **P1** should be comparable to that of the recycled catalyst **C1**. On the other hand, in the reaction with **S2**, a continuous change of the Pd species was observed (Figure 5b). An additional spectrum at 31 min was also collected and is shown in Figure S8 (Supporting Information). This spectrum is not included in Figure 5b due to the low signal-to-noise ratio; however, the features are very similar to that of the *ex situ* spectrum of recycled **C2**. This observation indicates that the transformation of the catalyst was close to complete after 31 min.^47^ It is intriguing that the reaction involving **S1** only produces a very small fraction of metallic Pd aggregates at 6 min and then ceases immediately, while with **S2**, the formation of metallic Pd aggregates occurs continuously and almost reaches completion.

As the change in the XANES spectra had already stopped after 6 min in the case of **S1**, our focus shifted toward studying the catalytic reaction with **S2**. After confirming that an extensive reduction had occurred, we were interested in determining the identity of the reducing agent. A control experiment was conducted in which the unused catalyst was suspended in toluene together with **S2** with neither TEA nor BQ present to see if any reduction occurred. The mixture was heated to 50 °C prior to data collection. The XANES spectra in Figure S9 (Supporting Information) show no significant features of metallic Pd after the edge, but a slight edge shift could be observed toward lower energy. The spectra are in good agreement with the *in situ* spectra in Figure 5a, indicating a similar composition of the Pd speciation. In another experiment, TEA was slowly introduced to the reactor, and the *in situ* XANES spectra recorded are shown in Figure S10 (Supporting Information). Here, the XANES spectra gradually transformed into the spectrum of metallic Pd during the continuous introduction of TEA, which clearly shows that TEA acts as a reducing agent.

**Figure 6:**
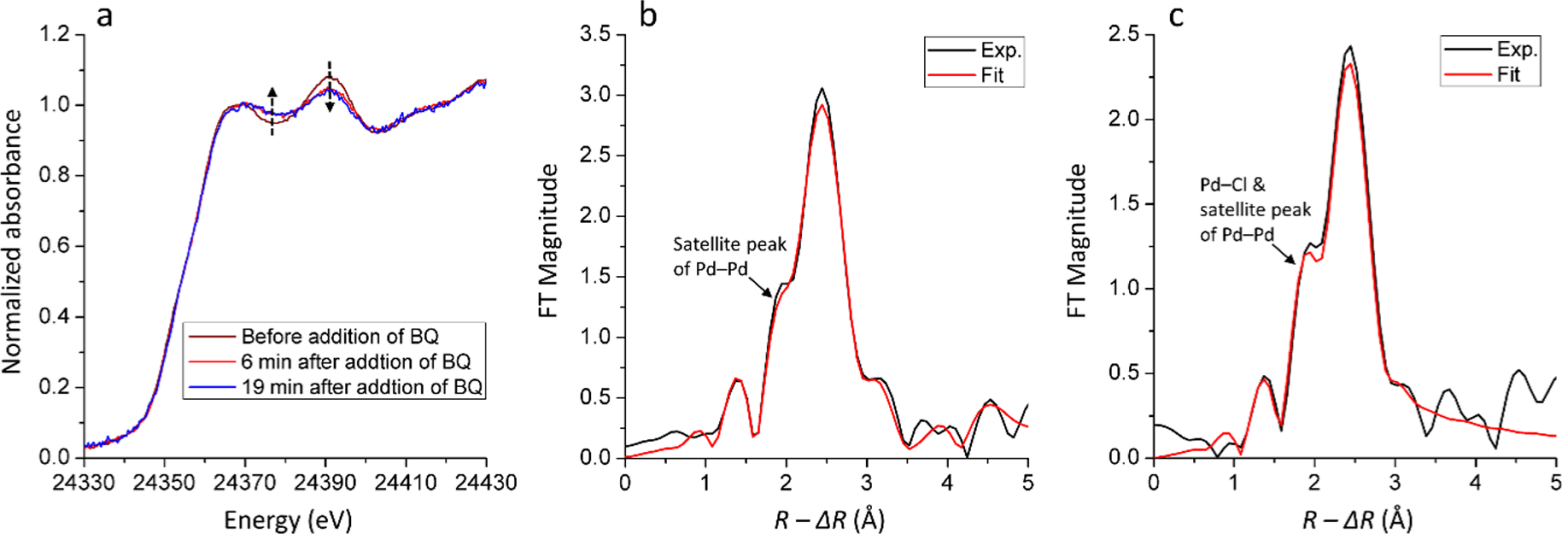
Representative *in situ* Pd K-edge (a) XANES spectra of the recycled catalyst from reaction with **S2**, (b) Fourier transformed *k*^3^-weighted EXAFS spectra of the recycled catalyst from reaction with **S2** before addition of BQ and (c) after the addition of BQ. The spectra in (b) and (c) are not phase-corrected and are Fourier transformed in the same *k* range, 2–10.5 Å^–1^.

The recycled catalyst **C2** was found to be essentially inactive after the first reaction cycle, but it was found that its catalytic activity could be restored by the addition of BQ. This observation made us interested in studying the BQ-promoted reactivation process by XAS. Figure 6 shows the XANES and FT-EXAFS spectra of the recycled catalyst from the reaction with **S2** before and after the addition of BQ to the Pd aggregates. The XANES spectra after the addition of BQ still exhibit features of metallic Pd aggregates. However, the amplitude of the XANES spectra decreased instantly after BQ was added, which suggests that BQ caused a decrease of the average size of the Pd aggregates.^12,44^ The FT-EXAFS spectra before and after the addition of BQ are compared in Figure 6b,c. The Pd–Pd distance was found to decrease slightly from 2.734(3) Å to 2.726(5) Å after adding BQ (see Table 1). This observation is in line with the changes of the XANES spectra that indicated a decreased average particle size, since it is known that a smaller particle size leads to a smaller mean coordination number and shorter mean bond distances.^46^ It should be noted that the shoulder peak at *ca*. 1.9 Å in Figure 6c (no phase correction) became slightly more pronounced compared to the main peak after BQ was added. The EXAFS refinement reveals that only Pd–Pd and Pd–N/O interactions are present in the first coordination shell of Pd before the addition of BQ (see Table 1). This observation indicates that the shoulder at around 1.9 Å belongs solely to the satellite peak of Pd–Pd single scattering, as marked in Figure 6b; however, any Pd–Cl interaction would be expected to appear at the same distance (see Figure 3a). The increased intensity of this shoulder at *ca*. 1.9 Å in Figure 6c compared to Figure 6b suggests that an additional scattering signal, other than the satellite peak of Pd–Pd single scattering, appeared. A single-scattering signal corresponding to Pd–Cl was unveiled upon performing an EXAFS refinement, and its average coordination number was determined to be *ca*. 0.6 (Table 1). As discussed above, any Cl left over from the synthesis of the Pd(II)-AmP-MCF or from leaching could potentially form Pd–Cl after the addition of BQ.

**Figure 7:**
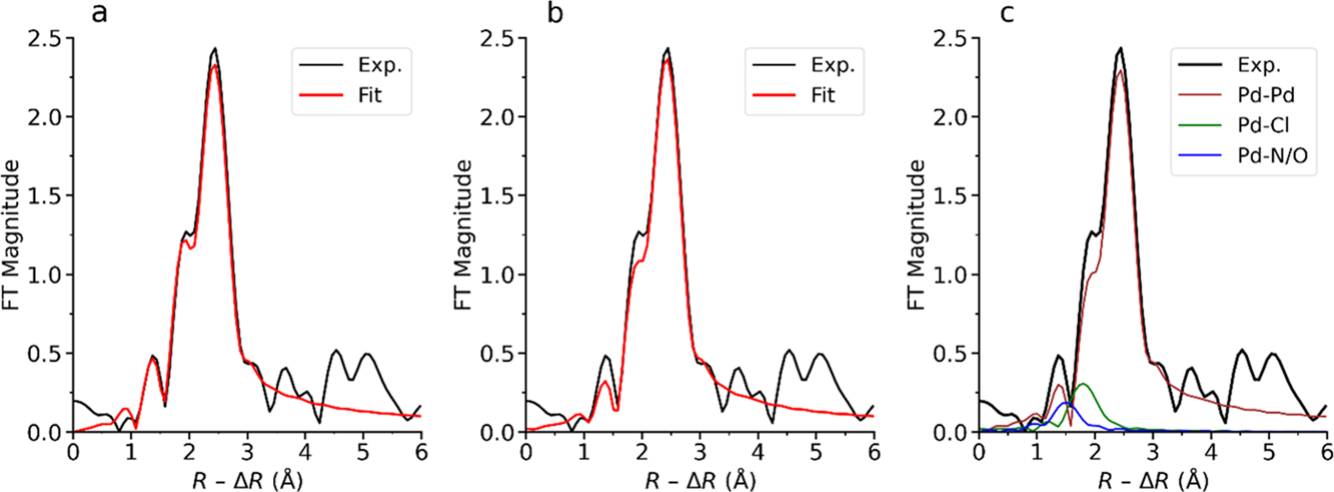
Fourier transformed *k*^3^-weighted EXAFS spectra of the recycled catalyst from reaction with **S2** after the addition of BQ (a) with and (b) without introducing Pd–Cl single scattering and (c) single-scattering components used in the fitting. The spectra are not phase-corrected and are Fourier transformed in the same *k* range, 2–10.5 Å^–1^.

An elaborate discussion on the coinciding positions of the Pd–Cl distance and the Pd–Pd single-scattering satellite peak can be found in a previous study.^11^ Using the same strategy, the fitting results with and without introducing Pd–Cl single scattering are compared and presented in Figure 7a,b, respectively. By introducing the single scattering of Pd–Cl, the fitting is noticeably improved, especially the shoulder at *ca*. 1.9 Å. Moreover, without introducing Pd–Cl, the distance of Pd–N/O is refined at 2.15(4) Å, which is significantly longer than a typical Pd–N/O distance. This is due to the fact that the software mathematically tries to compensate for the missing contribution at 2.3 Å in the fitting procedure. This comparison indicates the existence of Pd–Cl in the recycled catalyst after BQ was added. Moreover, Figure 7c shows the calculated single-scattering components of Pd–Pd, Pd–Cl, and Pd–N/O used in the fitting shown in Figure 7a to facilitate the understanding of the data. These observations provide experimental evidence for the reactivation of the recycled catalyst **C2**. The purpose of introducing BQ is to oxidize Pd(0) to Pd(II), and a reasonable scenario is that the surface atoms of the Pd aggregates become partially oxidized and bound to Cl^–^ ligands again, while the average size of the Pd aggregates becomes slightly smaller. Both these factors contribute to the recovery of the catalyst activity.

**Figure 8:**
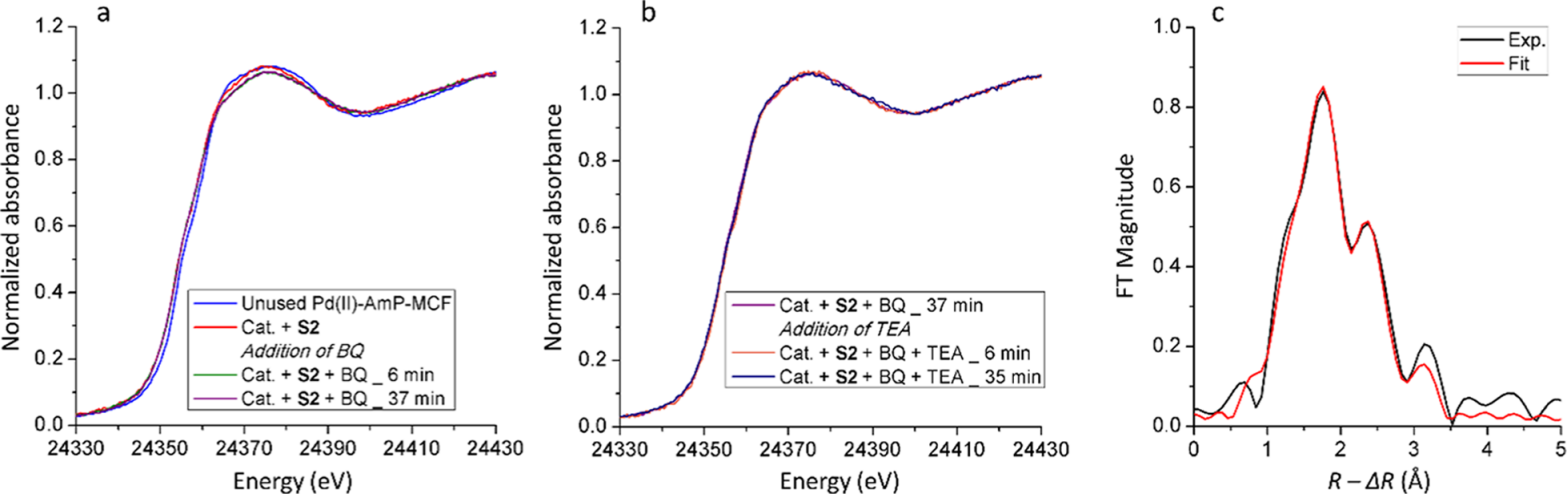
(a) Representative *in situ* Pd K-edge XANES spectra of Pd(II)-AmP-MCF after addition of **S2** and BQ and (b) continued measurements of the XANES spectra from Figure 6a after the addition of TEA. (c) Fourier transformed *k*^3^-weighted EXAFS spectrum of Pd(II)-AmP-MCF after the addition of BQ and TEA. The spectrum in Figure 6c is not phase-corrected and is Fourier transformed in the *k* range of 2–10 Å^–1^.

Through the elucidation of the mechanism of the catalyst deactivation and reactivation obtained from these XAS experiments, a new catalytic protocol with a significantly lower degree of deactivation could be designed. The key to prevent the deactivation process is to effectively suppress the transformation of the Pd(II) complexes into metallic Pd aggregates. This was achieved in practice by the addition of BQ before the start of the reaction and the addition of TEA at a later stage. The catalyst under these conditions was measured by *in situ* XAS, and the representative spectra are shown in Figure 8. The edge shifted slightly toward lower energy, and a minor change occurred in the spectrum after the edge when **S2** was added. Upon the addition of BQ, the edge position of the XANES spectrum remained the same, while the region after the edge further evolved slightly and then ceased (Figure 8a). The measurement was then continued while TEA was added, and the XANES spectra are shown in Figure 8b. The XANES spectra exhibited no changes even when TEA was added, and, moreover, no signs of further formation of metallic Pd aggregates were detected. The EXAFS spectrum of the catalyst after the addition of BQ was analyzed, and its Fourier transform is shown in Figure 8c with the primary refinement parameters summarized in Table 1. It should be emphasized that this minor formation of Pd aggregates is likely caused by **S2** and not by TEA. Most importantly, this serves as a confirmation of the validity of introducing BQ at the beginning of the reaction, which can effectively prevent any significant Pd reduction from occurring.

Furthermore, to obtain a more quantitative measure of the effectiveness of the reactivation strategy, we next set out to apply it to the recycled catalysts **C1** and **C2**, to see if it would be possible to boost their activity in a subsequent cycloisomerization reaction. The results from the recycling studies of Pd(II)-AmP-MCF with **S1** and **S2** under conventional BQ-free conditions are shown in Table 2.

**Table 2:**
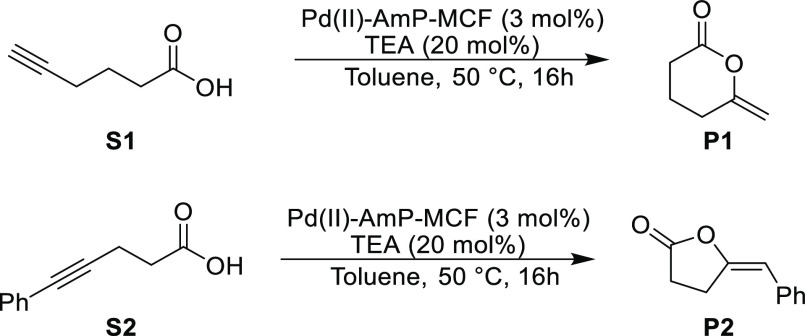
Recycling of the Catalyst with Substrates **S1** and **S2**^a^

entry	substrate	cycle	NMR yield (%)
1	**S1**	1	78
2	**S1**	2	74
3	**S1**	3	75
4	**S1**	4	26
5	**S1**	5	32
6	**S2**	1	42
7	**S2**	2	<5

a Reaction conditions: 0.4 mmol of **S1** or **S2**, 0.08 mmol of TEA, 0.012 mmol of Pd(II)-AmP-MCF, and 1 mL of toluene. The NMR yield was determined using 1,3,5-trimethoxybenzene as the internal standard.

When Pd(II)-Amp-MCF was used in the cyclization of **S1**, there was no significant loss of activity until the fourth cycle (Table 2, entries 1–5). This is consistent with the XAS result that showed that only a minor reduction occurred for recycled **C1**. However, in the cyclization of **S2**, essentially full deactivation was observed after the first cycle (entries 6 and 7).

To compare the catalytic activities of recycled **C1** and **C2** after one reaction cycle with their respective substrates, they were applied in the cyclization of a third substrate, **S3**, without the presence of BQ and using the standard cycloisomerization conditions reported in our previous study.^27^ Here, **C2** showed considerably lower activity than **C1** (Table 3, entries 1–2). In an attempt to reactivate the catalysts **C1** and **C2**, they were stirred with 1 mol % BQ before starting the reaction. Interestingly, the BQ-treated **C1** and **C2** were found to display significantly enhanced activity compared to the untreated catalyst (entries 3–4). Experiments were also performed where 1 mol % BQ was present from the beginning of the reaction (entries 5–6), and these experiments showed that the activities of both catalysts were excellently retained.

**Table 3:**
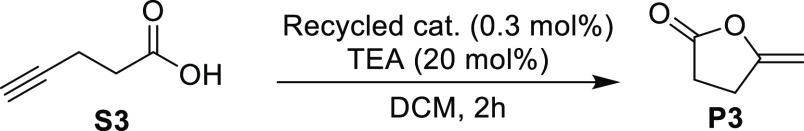
Cycloisomerization of **S3** Using Recycled **C1** and **C2**^a^

entry	catalyst	NMR yield (%)
1	**C1**	90
2	**C2**	77
3^b^	**C1**	99
4^b^	**C2**	95
5^c^	**C1**	>99
6^c^	**C2**	>99

a Reaction conditions: 0.4 mmol of **S3**, 0.08 mmol of triethylamine, 0.012 mmol of Pd(II)-AmP-MCF, and 1 mL of toluene. The NMR yield was determined using 1,3,5-trimethoxybenzene as the internal standard.

b 1 mol % BQ used to reactivate the catalyst before the reaction.

c 1 mol % BQ added at the beginning of the reaction.

Since having 1 mol % BQ from the beginning of the reaction was found to give the best result according to Table 3, these experiments were also performed with substrates **S1** and **S2** (Table 4). In the case of **S1**, full conversion to the product was observed for the first four cycles, and in the fifth cycle, a 95% yield of **P1** was obtained (entries 1–5). This should be compared with the results in Table 2 (entries 1–5) in the absence of BQ, where the first three cycles provided 75–78% of **P1** and cycles 4–5 gave <35% yield of **P1**. A similar dramatic improvement was observed for **S2** on addition of 1 mol % BQ. The first two cycles now gave a high yield of 82–85% of **P2** (Table 4, entries 6–7) to be compared with the 42% yield in the first cycle and <5% yield in the second cycle in the absence of BQ (Table 2, entries 6–7). From the third cycle, partial deactivation of the catalyst occurred with **S2**.

**Table 4:**
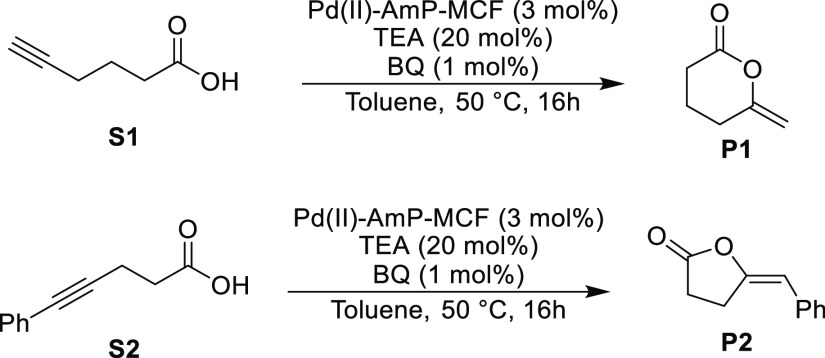
Recycling of the Catalyst with Substrates **S1** and **S2**^a^

entry	substrate	cycle	NMR yield (%)
1	**S1**	1	>99
2	**S1**	2	>99
3	**S1**	3	>99
4	**S1**	4	>99
5	**S1**	5	95
6	**S2**	1	85
7	**S2**	2	82
8	**S2**	3	57
9	**S2**	4	41
10	**S2**	5	28

a Reaction conditions: 0.4 mmol of **S1** or **S2**, 0.08 mmol of TEA, 0.012 mmol of Pd(II)-AmP-MCF, 0.0004 mmol of BQ, and 1 mL of toluene. The NMR yield was determined using 1,3,5-trimethoxybenzene as the internal standard.

The difference between the two substrates **S1** and **S2** with respect to the particle size of the corresponding recycled catalysts **C1** and **C2** (Figure 4) can be explained by the different coordination strengths of the two alkyne moieties (terminal versus internal). The terminal alkyne of **S1** coordinates more strongly to Pd(II) than the phenyl-substituted alkyne of **S2** due to steric reasons. The Pd(II) π-complex formed with **S2** is therefore not as stable as that formed with **S1**. Because of the stronger coordination of **S1** to palladium, Pd(II) would be expected to be protected from both reduction by TEA and aggregation to metallic Pd(0). On the other hand, Pd(II) is reduced to a larger degree when **S2** is used as the substrate, leading to larger Pd(0) aggregates. This difference between **S1** and **S2** would explain the overall smaller palladium particles in recycled **C1** in comparison to recycled **C2**, as shown by the STEM images (Figure 4).

One might argue that the active form of the catalyst could be isolated Pd atoms that have been leached from the heterogeneous scaffold. In our previous study, leaching was found to be a negligible 4 ppm according to ICP-OES.^27^ In a control experiment, Pd(OAc)_2_ was used as catalyst and a low yield of 18% was obtained under the same conditions. From these results, it can be safely concluded that the reaction is primarily catalyzed by the heterogeneous catalyst, although any Pd(II) species that might be produced from leaching or have been left over from the synthesis of the Pd(II)-AmP-MCF could in principle assist as well.

## Conclusions

In summary, we have introduced a new reactivation strategy for a heterogeneous Pd(II) catalyst that was found to be deactivated to a significant extent in our earlier work on the cycloisomerization of acetylenic acids.^18^ By adding 1 mol % BQ at the beginning of the reaction, it is possible to suppress this deactivation process and maintain high catalytic activity. The development of this strategy was supported by XAS investigations, which enabled the monitoring of the changes in the oxidation states and coordination environments of the Pd centers *in situ*. These XAS studies also provided experimental support for the hypothesis that the deactivation mechanism of the Pd(II)-AmP-MCF catalyst is due to the formation of catalytically inactive Pd(0) aggregates. Although TEA was found to be responsible for the reduction of the Pd(II)-centers, it was observed that the rate and the extent of this reduction process were affected by the choice of the acetylenic acid substrate.

This study has provided an in-depth analysis of the deactivation pathway for a heterogeneous Pd catalyst used in the cycloisomerization of acetylenic acids. The reaction was studied in real time using XAS, and the mechanistic insight gained from these spectroscopic studies was used to identify the cause of the deactivation, which in turn enabled the development of a new and more robust catalytic protocol. This deactivation process was shown to be caused by the reductive aggregation of palladium triggered by TEA. Interestingly, by maintaining oxidative conditions during the reaction by adding BQ before starting the reaction, deactivation could be efficiently suppressed. This reactivation strategy allowed for the recycling and reapplication of the heterogeneous catalyst through multiple reaction cycles.

## Supporting Information Available

General experimental and data analysis details; description of the *in situ* reactor; additional XANES and EXAFS spectra; and additional STEM images (PDF)
