# Supplementary material for: Investigation of the Deactivation and Reactivation Mechanism of a Heterogeneous Palladium(II) Catalyst in the Cycloisomerization of Acetylenic Acids by In Situ XAS
Source: ACS Catal. 2021 Feb 22;11(5):2999–3008. doi: 10.1021/acscatal.0c04374 (PMC8028044; doi:10.1021/acscatal.0c04374)
Supplement: Supplementary file 1 — cs0c04374_si_001.pdf [file cs0c04374_si_001.pdf]

Supporting information

Investigation of the Deactivation and Reactivation Mechanism of a Heterogeneous Palladium(II) Catalyst in the Cycloisomerization of Acetylenic Acids by *In Situ* XAS

Ning Yuan,[‡a,c] Arnar Guðmundsson,[‡,b] Karl P. J. Gustafson,[a,b] Michael Oschmann,[b] Cheuk-Wai Tai,[a] Ingmar Persson,[c] Xiaodong Zou,[a] Oscar Verho,[*,b,d] Éva G. Bajnóczi,[*,c,e] Jan-E. Bäckvall[*,b,f]

[a]Department of Materials and Environmental Chemistry, Arrhenius Laboratory, Stockholm University, SE-106 91 Stockholm, Sweden

[b]Department of Organic Chemistry, Arrhenius Laboratory, Stockholm University, SE-106 91 Stockholm, Sweden

[c]Department of Molecular Sciences, Swedish University of Agricultural Sciences, P.O. Box 7015, SE-750 07 Uppsala, Sweden

[d]Department of Medicinal Chemistry, Uppsala Biomedical Centre, Uppsala University, SE-751 23 Uppsala, Sweden

[e]Wigner Research Centre for Physics, H-1121 Budapest, Hungary

[f] Department of Natural Sciences, Mid Sweden University, Holmgatan 10, SE-851 70 Sundsvall, Sweden

[‡] These authors contributed equally to this work

*Correspondence should be addressed to: jeb@organ.su.se or bajnoczi.eva@wigner.hu or oscar.verho@ilk.uu.se

Contents

Section 1. General experimental details S1

Section 2. *In situ* reactor and XAS data analysis S4

Section 3. Additional XANES and EXAFS analysis S5

References S14

Section 1. General experimental details

General information

^{1}H NMR and ^{13}C NMR spectral data were recorded at 400 MHz and 100 MHz, respectively. Chemical shifts (δ) are reported in ppm, using the residual solvent peak in $CDCl_3$ (H = 7.26 and C = 77.0 ppm) as internal standard, and coupling constants (J) are given in Hz. HRMS were recorded using ESI-TOF techniques. Chemicals were purchased from commercial sources. Silica gel chromatography was performed either manually (particle size 40-63µm, pore size 60Å and mesh size 230-400) or on an automated flash machine equipped with a UV detector, with 12-40 g silica columns (particle size 40-63 µm, mesh size 230-400, pore size 60 Å). Reactions were monitored using aluminum-backed plates (1.5 Å, 5 cm) pre-coated (0.25 mm) with silica gel, UV light or potassium permanganate stain for visualization.

Synthesis of 5-phenylpent-4-ynoic acid (S2)

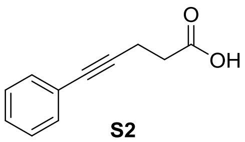

S2

The acetylinic acid **S2** was prepared through Sonogashira coupling using a reported procedure.[1] $Pd(PPh_3)_4$ (0.71 g, 0.61 mmol) and CuI (0.23 g, 1.22 mmol) were added to a dry round bottom flask. The system was argonized and iodobenzene (2.5 g, 12.2 mmol) was added. The solution was stirred at room temperature for 10 min. Et_3N (20 mL) and pent-4-ynoic acid (1.0 g, 10.2 mmol) were added and the mixture was stirred at room temperature for 12 h.
The reaction was diluted with EtOAc and water, and the aqueous layer was washed with EtOAc three times. The aqueous layer was acidified with HCl (2M) and extracted with EtOAc. The combined aqueous layers were dried with Na_2SO_4, filtered and concentrated under reduced pressure. **S2** was obtained as white/yellow crystals (90 %). Spectral data is in accordance with those reported in the literature.[2]

Synthesis of Pd(II)-Amp-MCF

Pd(II)-Amp-MCF (7-8 wt% Pd) was prepared by a previously reported procedure.[3] Triblock copolymer PEG-PPG-PEG Pluronic® P-123 (8 g) was dissolved in an aqueous HCl solution (1.6 M, 150 mL). 1,3,5-Trimethylbenzene (6.8 mL) was subsequently added to the mixture and the resulting emulsion was stirred vigorously at 37-40 °C for 2 h. Tetraethoxysilane (18.4 mL) was then added to the reaction and it was allowed to stir for an additional 5 min. The resulting solution was then aged for 20 h at 40 °C under static conditions. NH_4F (92 mg) dissolved in H_2O (10 mL) was added and the reaction was stirred for 10 minutes before it was sealed, heated to 100 °C and allowed to age further for 24 h. The solutions was allowed to reach room temperature and then filtered. The resulting MCF precipitate was extensively washed with H_2O (4 L) and EtOH (2 L). The resulting powder was dried under vacuum for 24 hours before being calcinated at 560 °C in air for 6 h to remove the organic precursors and other impurities.

The unfunctionalized MCF material was dried for 4 h and then 40 mL of dry toluene were added. The resulting mixture was stirred under argon until a homogeneous suspension was obtained. (3-aminopropyl)trimethoxysilane (5.6 mL) in dry toluene (20 mL) was added to the mixture, the mixture was stirred at room temperature for 5 minutes and then at 110 °C for 48 h. The mixture was cooled to room temperature, filtered and washed with toluene (400 mL), EtOH (250 mL) and DCM (500 mL). The solid was then resuspended in EtOH (40 mL) and stirred at 60 °C for 16 h to remove unreacted

silane species. The mixture was filtered, washed with EtOH (250 mL) and DCM (250 mL) and dried over night by vacuum to give the functionalized AmP-MCF material.

Synthesis of 6-methylenetetrahydro-2*H*-pyran-2-one (P1)

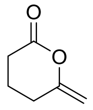

P1

5-Hexynoic acid (45 mg, 0.40 mmol), triethylamine (0.10 mmol), Pd(II)-AmP-MCF (3 mol%, 16.8 mg) and toluene (1 mL) were placed in a microwave vial, which was sealed and heated in an oil bath at 50 °C for 16 hours. The reaction mixture was then filtered through a plug of silica with pentane/EtOAc (3/2) as eluent. The solvent was removed *in vacuo* to afford the desired product. Spectral data is in accordance with those reported in the literature.[2]

Synthesis of 5-benzylidenedihydrofuran-2-(3*H*)-one (P2)

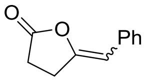

P2

5-phenylpent-4-ynoic acid (**2**, 70 mg, 0.40 mmol), triethylamine (0.10 mmol), Pd(II)-AmP-MCF (3 mol%, 16.8 mg) and toluene (1 mL) were placed in a microwave vial, which was sealed and heated in an oil bath at 50 °C for 16 hours. The reaction mixture was then filtered through a plug of silica with pentane/EtOAc (3/2) as eluent. The solvent was removed *in vacuo* to afford the desired product. Spectral data is in accordance with those reported in the literature.[2]

Synthesis of 5-methylenedihydrofuran-2(3*H*)-one (P3)

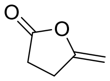

P3

Pent-4-ynoic acid (**S3**, 0.80 mmol), triethylamine (0.20 mmol), Pd(II)-AmP-MCF (0.30 mol%, 5.12 mg) and DCM (2 mL) were placed in a microwave vial, which was sealed and heated in an oil bath at 40 °C for 2 hours. The reaction mixture was then filtered through a plug of silica with pentane/EtOAc (3/2) as eluent. The solvent was removed *in vacuo* to afford the desired product. Spectral data is in accordance with those reported in the literature.[2]

XAS Experiments

All XAS data were collected at beamline P64 at the Petra III Extension, Deutsches Elektronen-Synchrotron (DESY), Hamburg, Germany. Pd K-edge (24.350 keV) was chosen with a range of 24.00 to 25.00 keV.[4] XAS measurements were performed in transmission mode. In addition to measurements of the catalyst, a palladium foil was measured at the same time and its first inflection point on the absorption edge was used to calibrate the XAS spectra. The unused and recycled catalysts were measured using standard sample holders for solid powers. A custom-made batch reactor was used for the *in situ* measurements, in which the catalytic reactions and control experiments were conducted. The scan time of one full XAS spectrum was set at ca. 6 min with a consideration of the data quality and time resolution. Detailed illustrations of the reactor, as well as the XAS data analysis can be found at Section S2.

Catalytic reaction condition for *in situ* XAS measurements

Cyclization of S1

Hex-5-ynoic acid (**S1**, 314 µL, 2.85 mmol), triethylamine (100 µL, 0.7 mmol), Pd(II)-AmP-MCF (120 mg), and toluene (2 mL) were added to the reaction shown in Figure S1 and the reaction was run at 50 °C.

Cyclization of S2

5-phenylpent-4-ynoic acid (**S2**, 483 mg, 2.85 mmol), triethylamine (100 µL, 0.7 mmol), Pd(II)-AmP-MCF (120 mg) and toluene (2 mL) were added to the reaction shown in Figure S1 and the reaction was run at 50 °C.

Section 2. *In situ* reactor and XAS data analysis

An *in situ* reactor, named *SynRAC*, was designed for solvothermal reactions.[5] The reaction vessel is a glass vial with 1 mm thick walls and 10 mm inner diameter. The maximum volume of the glass vial is 6 mL. The reaction mixture can be homogenized through magnetic stirring. It can also be heated by the heating mantle and the temperature can be monitored by immersing a thermal couple into the reaction mixture. The target temperature can be typically reached within 1 min. Additional reagents can be injected into the reactor remotely via injection tubes which are also connected to a neMESYS syringe pump. The core parts of the reactor shown in Figure S1 aligned on the beamline to allow the beam transmit through the samples.

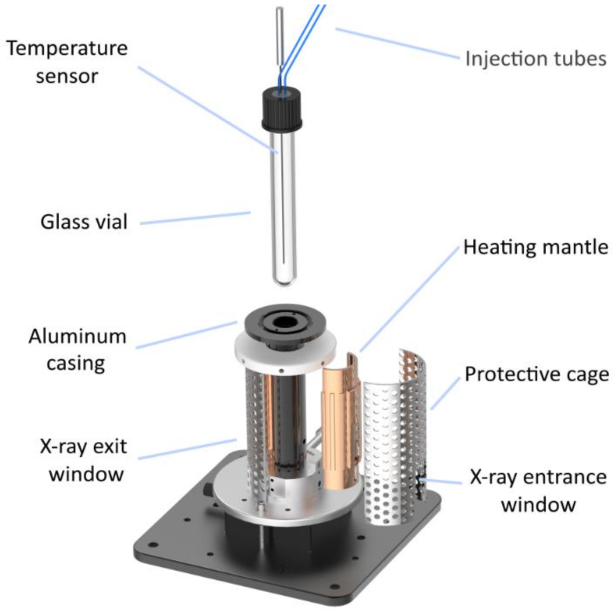

Figure S1. Custom-made reactor *SynRAC* for *in situ* XAS. Reprinted with permission from *J. Am. Chem. Soc*. **2018**, *140*, 8206-8217. Copyright (2018, American Chemical Society)

XAS data were basically divided into two parts: XANES and EXAFS. To facilitate comparison of the XANES spectra, they were calibrated individually by a Pd foil reference, and normalized in the same way. EXAFSPAK package was used to analyse all the spectra including pre-edge subtraction, spline removal and Fourier transformation.[6] k^3-weighted EXAFS spectra were extracted and analysed. Non-linear least-squares fits were applied to compare with the experimental data. The model parameters

including number of backscattering atoms (*N*), mean interatomic distances (*d*), Debye-Waller factor coefficients (σ^2), many-body amplitude reduction factor (S_0^2), and threshold energy (E_o) were refined. For a given run of fitting, *N* was always fixed, while *d*, σ^2 and S_0^2 were refined. The *ab initio* program package FEFF7 was used to calculate the theoretical phases and amplitudes.[7] The standard deviations reported for the refined parameters were obtained from k^3- weighted least-squares refinements of the EXAFS function $\chi(k)$, and without including systematic errors. For a well-defined interaction, the accuracy of the distances given for an individual complex is between ±0.005 and ±0.02 Å.

Section 3. Additional XANES and EXAFS analysis

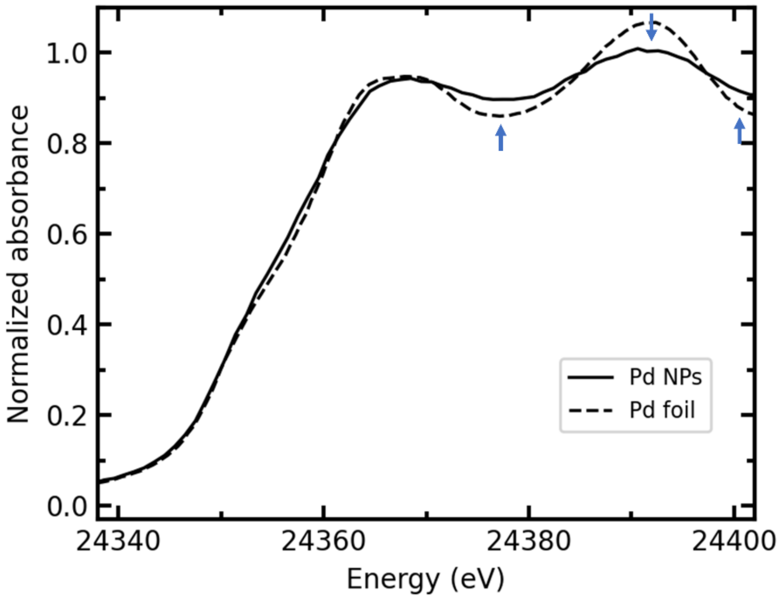

Figure S2. Pd K-edge XANES spectra of Pd nanoparticle measured in He flow and Pd foil.

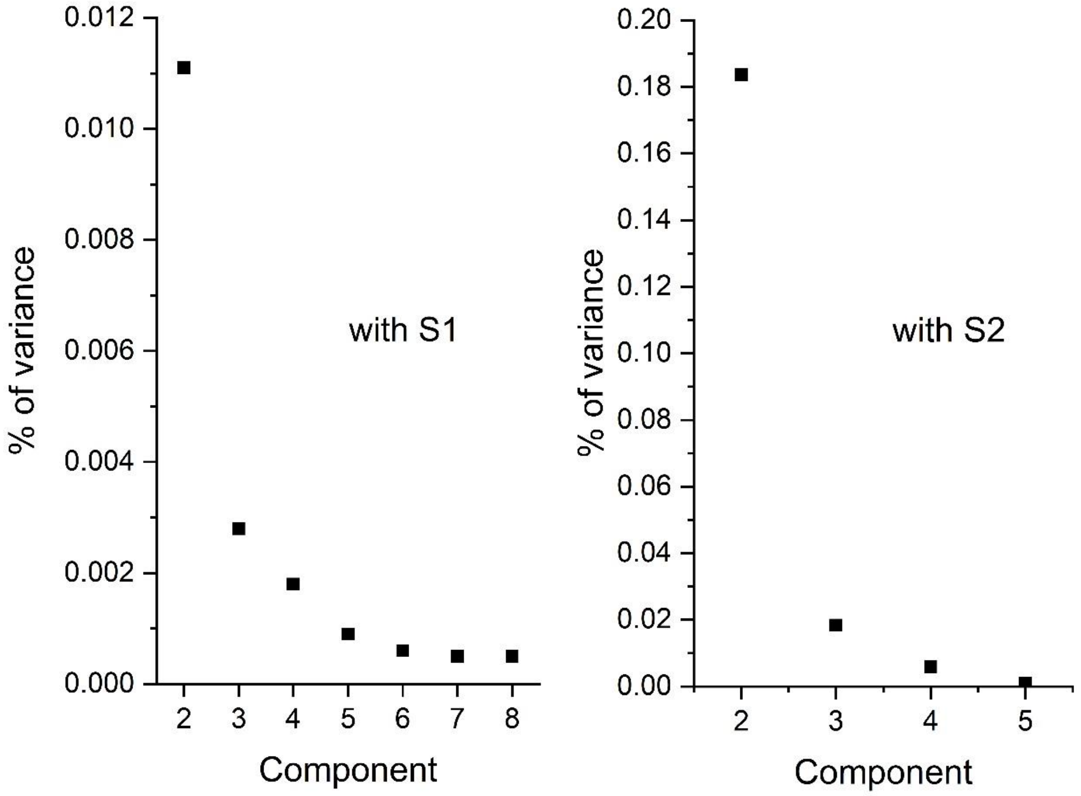

Figure S3. Scree plot of the principal component analysis of Pd K-edge XANES spectra of (left) recycled **C1** and (right) recycled **C2**.

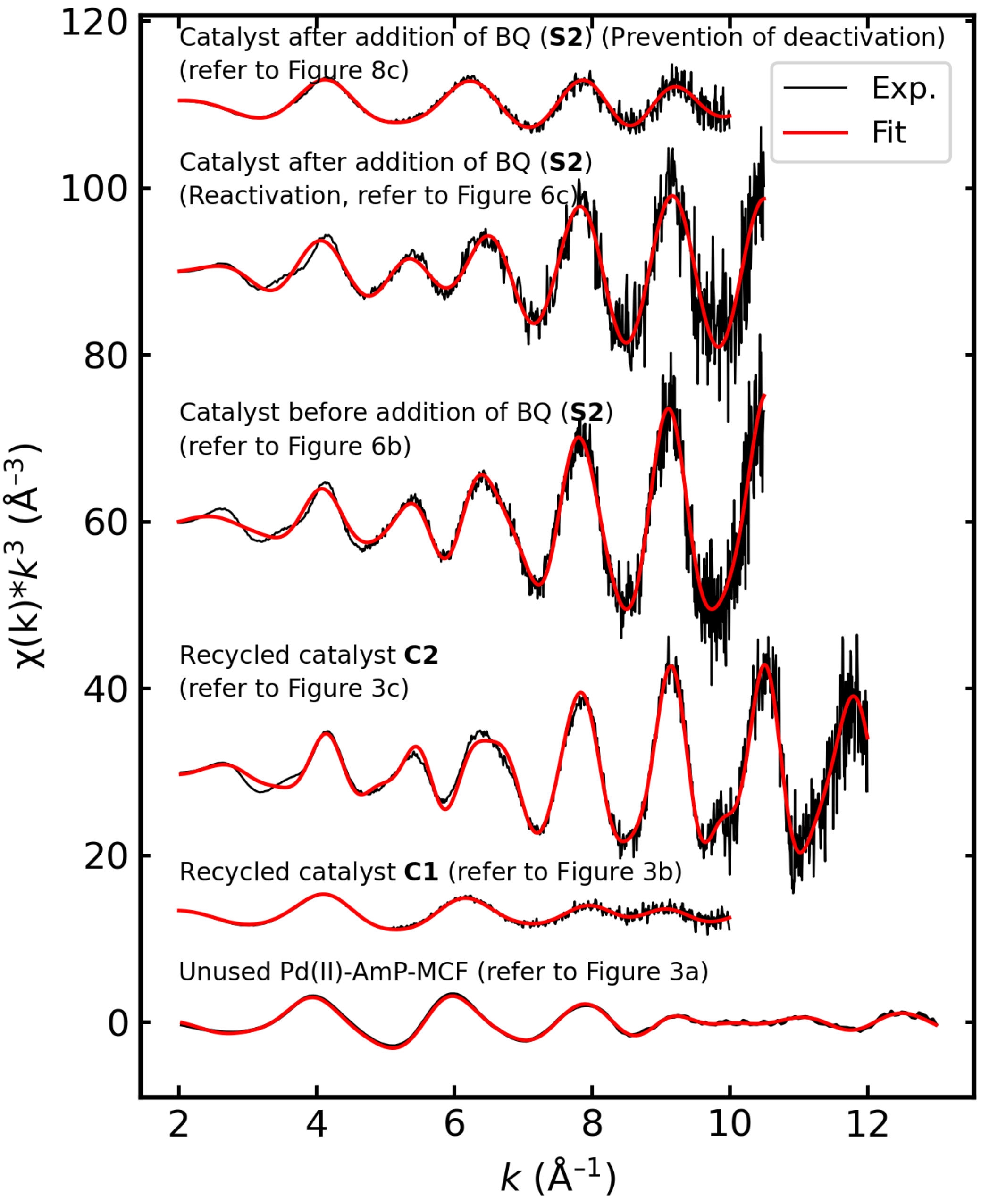

Figure S4. Experimental and fitting of k^3-weighted EXAFS spectra of the Pd(II)-AmP-MCF at different conditions. The selected fitting parameters can be found in Table. 1.

Table S1. Values of reduced error, ΔE_o and the *k*-range used for the best fits shown in Table 1 in the manuscript.

**Sample**	**Reduced error (chi-squared)**	**ΔE_o**	***k*-range (Å^{-1})**
Pd(II)-AmP-MCF (unused)	0.044	-18.6(3)	2–13
Recycled **C1**	0.15	-12.0(6)	2–10
Recycled **C2**	4.20	-7.5(4)	2–12
Catalyst before addition of BQ (**S2**)	5.85	-9.9(7)	2–10.5
Catalyst after addition of BQ (re-activation, **S2**)	7.51	-10(1)	2–10.5
Catalyst after addition of BQ (prevention of deactivation, **S2**)	0.58	-11.6(9)	2–10

Table S2. Coordination number, ***CN***, mean distances, ***d*** (Å), and Debye-Waller coefficients, σ^2 (Å^2), many-body amplitude reduction factor, S_0^2, in the EXAFS studies of the catalyst at different conditions. Parameters of all scattering paths including outer coordination shells and multiple scattering (MS) are included.

Samples	***Interaction***	***CN^a***	***d (Å)***	***σ^2 (Å^2)***	***S_0^2***
Pd(II)-AmP-MCF	Pd–N	2.0	2.023(2)	0.0035(3)	0.93(2)
	Pd–Cl	2.0	2.294(2)	0.0058(2)	
	Pd···Pd	0.5	3.065(5)	0.0083(4)	
	Pd···Pd	1.0	3.415(6)	0.0094(4)	
	MS ($PdCl_2$)	2.0	4.59(2)	0.007(2)	
*Recycled **C1***	Pd–N/O	2.0	2.034(4)	0.0032(7)	0.93(5)
	Pd–Cl	0.8	2.337(5)	0.0035(9)	
	Pd–Pd	0.5	2.72(1)	0.011(2)	
	Pd···Pd	1.0	3.44(1)	0.013(1)	

*Recycled **C2***	Pd–N/O	0.5	2.07(3)	0.004(3)	0.92(4)
	Pd–Pd	8.0	2.741(2)	0.0062(2)	
	Pd···Pd	4.0	3.846(9)	0.0114(9)	
	Pd···Pd	15.0	4.766(4)	0.0096(4)	
*Catalyst before addition of BQ (**S2**)*	Pd–N/O	1.0	2.05(1)	0.003(2)	0.92(8)
	Pd–Pd	7.0	2.734(3)	0.0042(6)	
	Pd···Pd	3.0	3.82(1)	0.005(1)	
	Pd···Pd	0.5	3.31(3)	0.002(3)	
	Pd···Pd	14.0	4.738(8)	0.0112(8)	
*Catalyst after addition of BQ (re-activation, **S2**)*	Pd–N/O	0.6	2.05(4)	0.002(6)	0.9(1)
	Pd–Cl	0.6	2.35(3)	0.004(4)	
	Pd–Pd	7.0	2.726(5)	0.0064(8)	
*Catalyst after addition of BQ (prevention of deactivation, **S2**)*	Pd–N/O	1.5	2.011(8)	0.002(2)	0.84(7)
	Pd–Cl	1.5	2.303(6)	0.003(1)	
	Pd–Pd	1.5	2.731(6)	0.0075(7)	
	Pd···Pd	1.0	3.44(2)	0.011(2)	

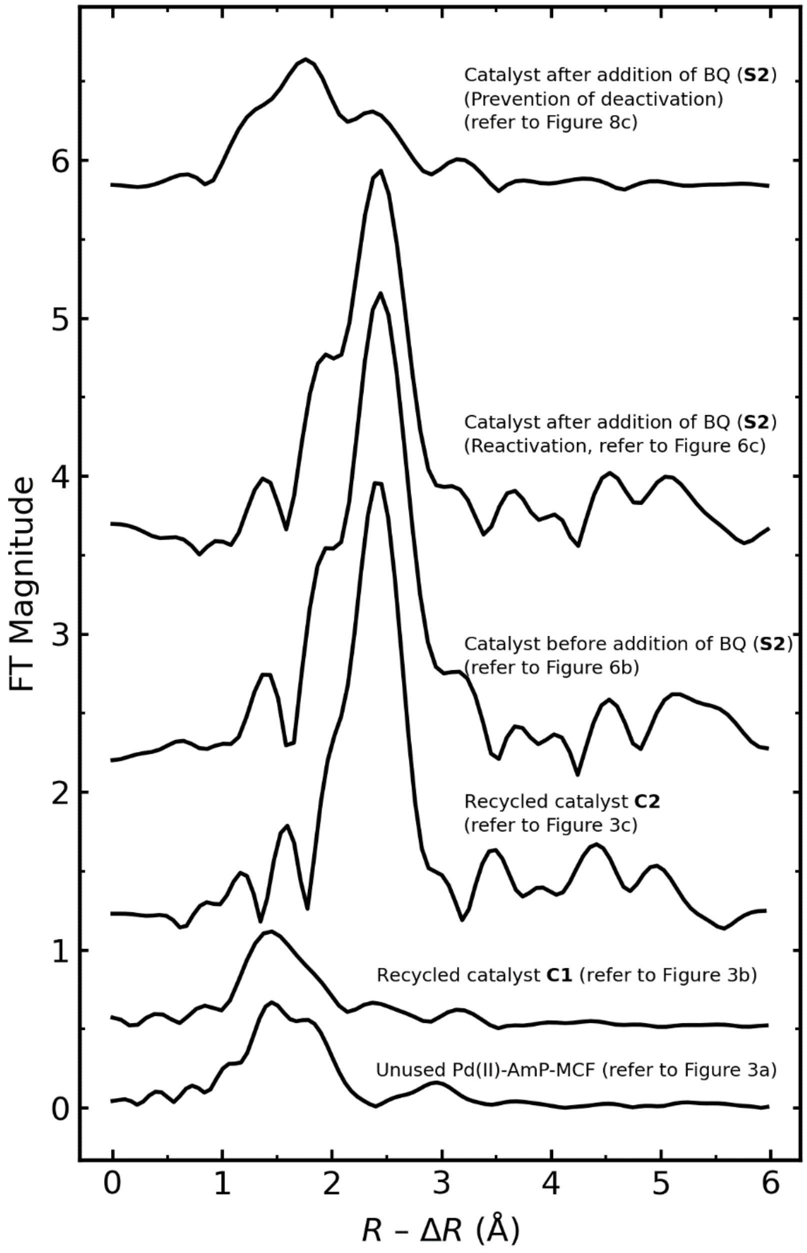

Figure S5. Fourier transformed k^3-weighted EXAFS spectra of the Pd(II)-AmP-MCF at different conditions. The selected fitting parameters can be found in Table. 1.

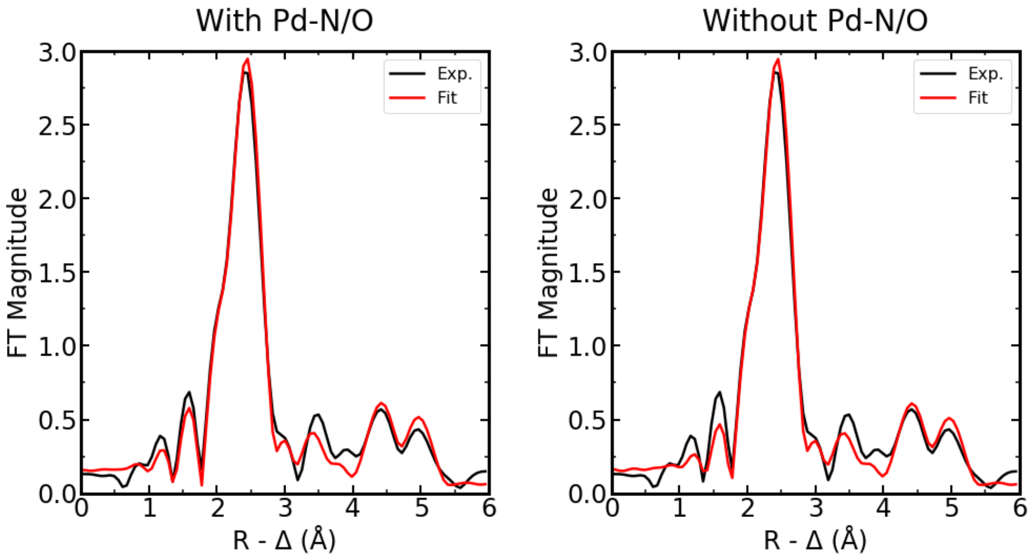

Figure S6. Fourier transformed k^3-weighted EXAFS spectra of the recycled catalyst **C2** with (reduced χ^2 = 4.20) and without (reduced χ^2 = 4.27) introducing Pd–N/O single scattering in the refinements. The spectra are not phase corrected. The k ranges used to perform Fourier transform 2–12 Å^{-1}, respectively.

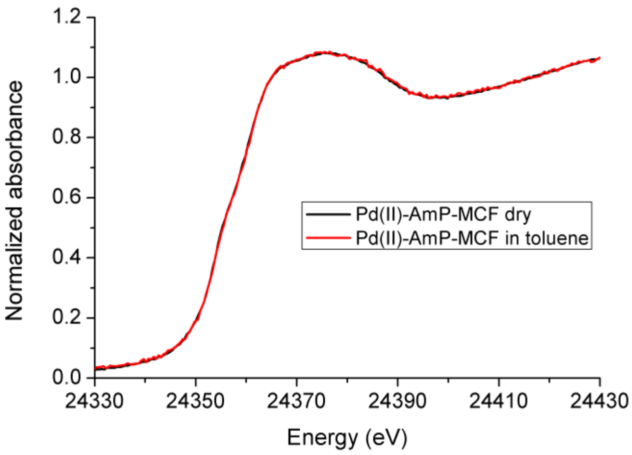

Figure S7. Pd K-edge XANES spectra of the Pd(II)-AmP-MCF in dry form and in toluene.

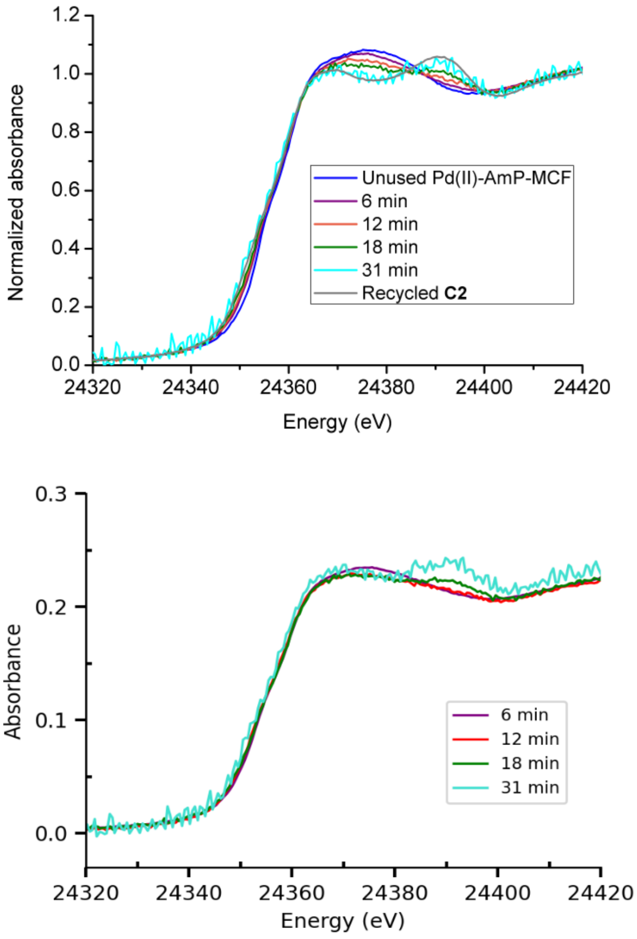

Figure S8. Pd K-edge XANES spectra of the Pd(II)-AmP-MCF when catalyzing **S2**. The XANES spectra of the as-synthesized and recycled catalysts are included. (Top) Normalized spectra. (Bottom) Unnormalized spectra.

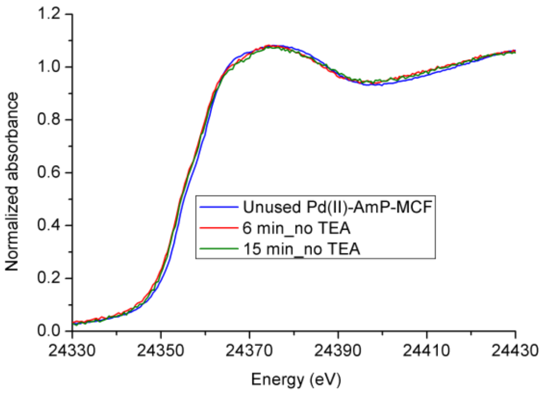

Figure S9. Pd K-edge XANES spectra of the Pd(II)-AmP-MCF when mixing with toluene and **S2**, and without TEA and BQ.

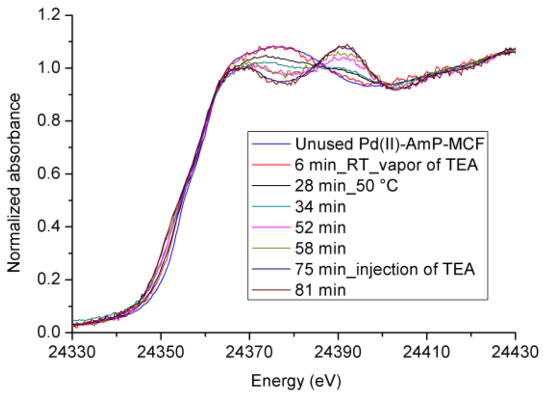

Figure S10. Pd K-edge XANES spectra of the Pd(II)-AmP-MCF when slowly introducing TEA into the reaction mixture.

Section 4. STEM Experiments and additional images

STEM Experiments

Scanning transmission electron microscopy (STEM) study was performed by a JEOL JEM-2100F microscope operated at 200 kV and equipped with a Schottky field-emission gun. Bright-field (BF) and high-angle annular dark-field (HAADF) STEM images were simultaneously acquired by Gatan BF and JEOL ADF detector, respectively, at the camera length of 8 cm. The sample was crushed in a mortar and then deposited on a TEM copper grid with holey carbon supporting films.

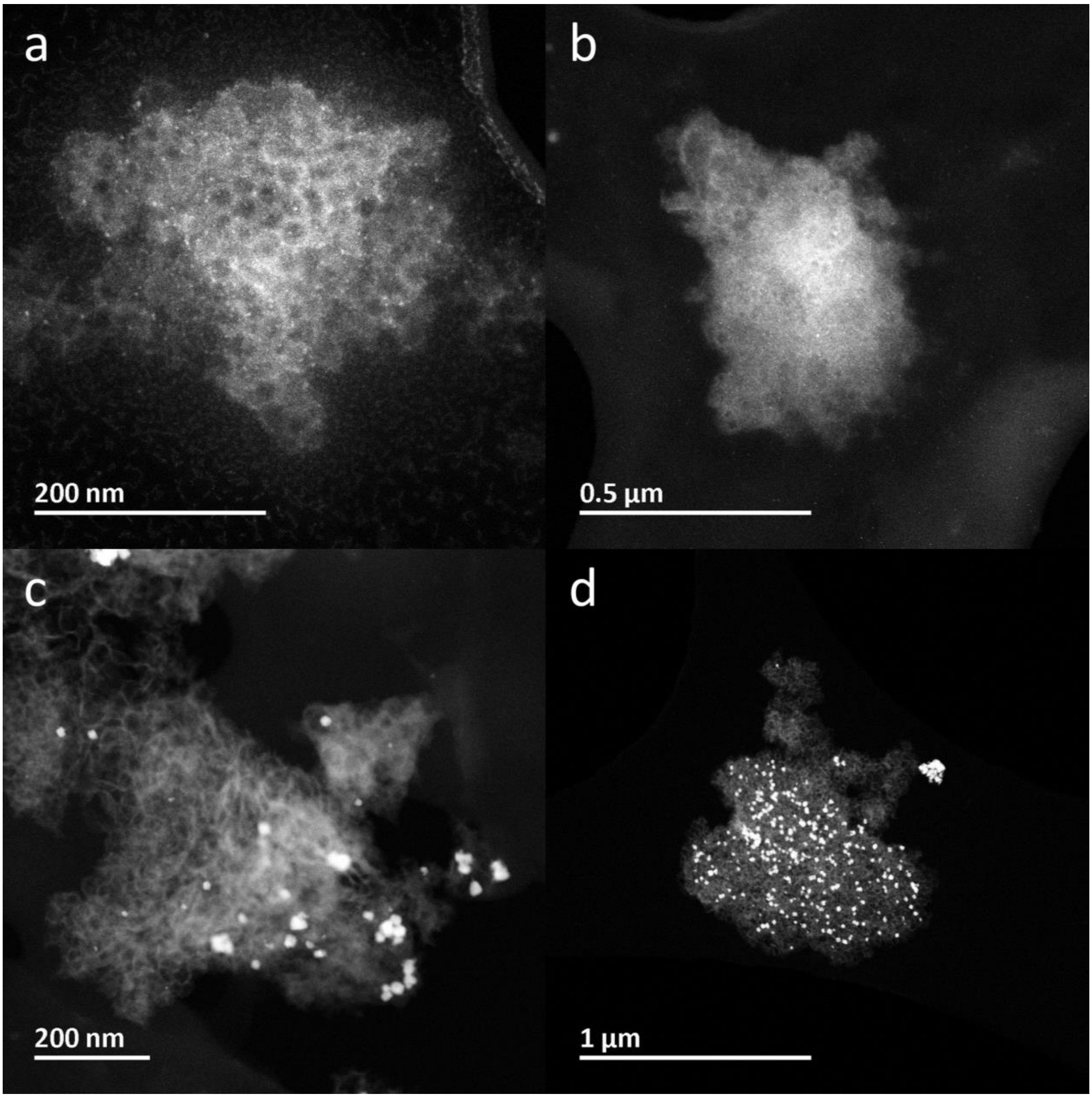

Figure S11. HAADF-STEM images of the recycled **C1** (a-b) and **C2** (c-d) showing the Pd nanoparticles (in white).

References

(1) Wu, H.; He, Y-P.; Gong, L-Z. *Adv. Synth. Catal.* **2012**, *354*, 975-980.

(2) Nagendiran, A.; Verho, O.; Haller, C.; Johnston, E. V. Bäckvall, J.-E. J. Org. Chem. 2014, 79, 1399-1405.

(3) Verho, O.; Åkermark, T.; Johnston, E. V.; Gustafson, K. P. J.; Tai, C-W.; Svengren, H.; Kärkäs, M. D.; Bäckvall, J.-E. Åkermark, B. *Chem. Eur. J.* **2015**, *21*, 5909-5915.

(4) Thompson, A.; Attwood, D.; Gullikson, E.; Howells, M.; Kim, K.-J; Kirz, J.; Kortright,

J.; Lindau, I.; Liu, Y.; Pianetta, P.; Robinson, A.; Scofield, J.; Underwood, J.; Williams, G.; Winick, H. *X-ray data booklet*, 3rd edn., Lawrence Berkley National Laboratory, **2009**.

(5) N. Heidenreich, U. Rütt, M. Köppen, A. K. Inge, S. Beier, A.- C. Dippel, R. Suren and N. Stock, *Rev. Sci. Instrum.*, 2017, **88**, 104102.

(6) G. N. George, I. J. Pickering, EXAFSPAK – A Suite of Computer Programs for Analysis of X-ray Absorption Spectra, SSRL, Stanford, CA. 1993.

(7) S. I. Zabinsky, J. J. Rehr, A. Ankudinov, R. C. Albers, M. J. Eller, *Phys. Rev. B* 1995, **52**, 2995–3009.
